# Sexual Functioning, Desire, and Satisfaction in Women with TBI and Healthy Controls

**DOI:** 10.1155/2015/247479

**Published:** 2015-10-18

**Authors:** Jenna Strizzi, Laiene Olabarrieta Landa, Monique Pappadis, Silvia Leonor Olivera, Edgar Ricardo Valdivia Tangarife, Inmaculada Fernandez Agis, Paul B. Perrin, Juan Carlos Arango-Lasprilla

**Affiliations:** ^1^Department of Psychology, University of Almeria, Almeria, Spain; ^2^Department of Psychology, University of Deusto, Bilbao, Spain; ^3^Division of Rehabilitation Sciences, School of Health Professions, The University of Texas Medical Branch, Galveston, TX, USA; ^4^Brain Injury Research Center, TIRR Memorial Hermann, Houston, TX, USA; ^5^Grupo de Investigación Carlos Finlay, Facultad de Salud, Universidad Surcolombiana, Neiva, Colombia; ^6^Department of Psychology, Virginia Commonwealth University, Richmond, VA, USA; ^7^Basque Foundation for Science (Ikerbasque), Bilbao, Spain

## Abstract

Traumatic brain injury (TBI) can substantially alter many areas of a person's life and there has been little research published regarding sexual functioning in women with TBI. *Methods*. A total of 58 women (29 with TBI and 29 healthy controls) from Neiva, Colombia, participated. There were no statistically significant differences between groups in sociodemographic characteristics. All 58 women completed the Sexual Quality of Life Questionnaire (SQoL), Female Sexual Functioning Index (FSFI), Sexual Desire Inventory (SDI), and the Sexual Satisfaction Index (ISS). *Results*. Women with TBI scored statistically significantly lower on the SQoL (*p* < 0.001), FSFI subscales of desire (*p* < 0.05), arousal (*p* < 0.05), lubrication (*p* < 0.05), orgasm (*p* < 0.05), and satisfaction (*p* < 0.05), and the ISS (*p* < 0.001) than healthy controls. Multiple linear regressions revealed that age was negatively associated with some sexuality measures, while months since the TBI incident were positively associated with these variables. *Conclusion*. These results disclose that women with TBI do not fare as well as controls in these measures of sexual functioning and were less sexually satisfied. Future research is required to further understand the impact of TBI on sexual function and satisfaction to inform for rehabilitation programs.

## 1. Introduction

Sexuality and sexual functioning are important aspects of one's life experience. Greater sexual dysfunction among women (43%) compared to men (31%) has been reported in the general population [1]. The disruption of brain functioning as a result of a traumatic brain injury may cause changes to the endocrine system [2]. In addition, sexual functioning may also be impacted by the well-documented physical, emotional, cognitive, behavioral, and relationship changes after TBI [3]. Despite the limited research on sexual dysfunction after TBI, the sample percentage reporting of sexual dysfunction has ranged in some studies from 29 to 54%, depending on the assessment time frame [4–7]. Diverse sampling methods, small sample sizes, and time of assessment after injury may contribute to differing reports of sexual dissatisfaction after TBI.

To date, only five studies have conducted a comparative analysis of sexual functioning between persons with TBI and healthy controls [4, 5, 8–10]. Overall, persons with TBI in these studies reported greater sexual dysfunction in comparison to healthy controls. Although some studies on sexual dysfunction after TBI have included women, few studies have specifically researched sexual dysfunction in women [7–9, 11].

One study found that women with TBI, having both an endocrine disorder and depression, were the most sensitive predictors of sexual difficulties [8]. Also, women with TBI in comparison to women without disability have reported greater sexual dysfunction in areas, such as sexual energy, sexual drive, ability to achieve orgasm, and sexual arousal [8]. However, two studies did not find gender differences in sexuality between men and women [9, 11]. It appeared that men and women identified similar negative changes regarding sexuality and sexual functioning following TBI. Gaudet and colleagues [9] reported that men with TBI had great sexuality concerns compared to women with TBI as well as men and women without TBI; however, their sample size was smaller. Another study made comparisons using community-based norms. Women with TBI at one year following injury reported reduced sexual cognition/fantasy, sexual arousal, sexual behavior/experience, and orgasm [7]. In addition, when compared to men, women with TBI reported significantly lower sexual cognition/fantasy and sexual arousal, which may be due to disruptions in endocrine functioning and emotional functioning [12].

Despite the importance of sexuality after TBI and the unique issues that women with TBI experience in terms of sexual dysfunction, research efforts have utilized nonequivalent control groups, community-based norms, and nonstandardized measures of sexual functioning, which are methodological limitations in the assessment of sexuality after TBI. Several demographic and injury-related characteristics related to sexuality have been identified in the TBI literature, such as age [8, 11, 13–15], time after injury [3, 13, 14], and injury severity [3]. However, the impact of these factors on sexual functioning in women with TBI is missing from the literature. As a result, the aims of this study were (1) to compare the sexual functioning, desire, and satisfaction of women with TBI to a comparison group and (2) to investigate the association between age, time after injury, and injury severity and sexual functioning, desire, and satisfaction.

## 2. Methods

### 2.1. Participants

Thirty-nine Spanish-speaking Colombian women with a moderate to severe TBI were identified in July of 2013 through a systematic review of all medical records in Hospital Universitario Hernando Moncaleano Perdomo de Neiva from August 2012 to March 2013. All patients had moderate to severe TBI confirmed in these medical records (loss of consciousness, positive computerized tomography, or magnetic resonance imaging). Inclusion criteria required that each participant be between the ages of 18 and 65, and participants were excluded if they had a history of neurological or psychiatric conditions, alcohol or drug abuse, or learning disabilities. All 39 met these inclusion criteria. Between April and December of 2013, these patients were contacted and invited to participate in the study when at least six months had passed since their injury. Ten women could not be contacted and the other 29 consented and completed the study.

The sample of twenty-nine women with TBI had an average age of 36.41 years (SD = 11.87), the average years of education were 9.41 (SD = 3.35), the mean time since injury was 18.03 months (SD = 10.41), and the mean Glasgow Coma Scale score at the time of admission to the hospital was 8.07 (SD = 3.80). The majority (44%) sustained their TBI in a motorcycle accident, 35% were in cohabiting relationships at the time of participation, 35% were employed full-time, and 97% of the TBI sample were self-identified as heterosexual.

For every female patient who completed the study, a healthy female control matched for age was identified and recruited. All controls approached did not have a history of TBI, neurological or psychiatric conditions, alcohol or drug abuse, or learning disabilities. All consented and completed the study. The healthy control group was comprised of 29 women, with an average age of 36.34 years (SD = 11.97). At the time of study participation, 31% were single and employed full-time, and the majority of controls (97%) were self-identified as heterosexual. There were no statistically significant differences based on age, education, marital status, or employment status between the control group and the TBI group (see Table 1).

### 2.2. Measures

#### 2.2.1. Female Sexual Function Index (FSFI)

The FSFI is a self-report measure of female sexual function, which contains 19 items. It has 6 domains: desire (2 questions), arousal (4 questions), lubrication (4 questions), orgasm (3 questions), satisfaction (3 questions), and pain (3 questions) [16]. The full-scale score is obtained by adding the six domain scores, and a score of zero indicates that no sexual activity was reported during the past month. The domain scores and total score can be derived with the following formula. For individual domain scores, items that comprise the domain are added. Then, the sum is multiplied by the domain factor (ranging from 0.3 to 0.6). The total score is the sum of six domains scores, which range from 2 to 36. Higher scores indicate greater sexual function. It has excellent psychometric properties and it supports the clinical utility [17–20]. The Spanish version was used which had been previously validated by Blumel et al. [21].

#### 2.2.2. Index of Sexual Satisfaction (ISS)

The ISS is a self-report scale that measures the degree of dissatisfaction in the sexual component of a dyadic relationship [22]. It contains 25 items [22], and scores range from 0 to 100 in which higher scores indicate greater sexual dissatisfaction. The ISS has a clinical cutoff score of 30 such that scores above that value indicate the presence of a clinically significant degree of sexual discord in the relationship. It has been used in clinical samples [23–25] and has shown good psychometric properties; the validated Spanish version was used in this study [26].

#### 2.2.3. Sexual Quality of Life Questionnaire (SQoL)

The SQoL has male (SQoL-M) and female (SQoL-F) forms. It evaluates the impact of sexual dysfunction on quality of life, including sexual confidence, emotional well-being, and relationship issues. The SQoL-M has 18 items and the SQoL-F has 18 items, each with a 6-point response scale (“completely agree” to “completely disagree”), and standardized total scores range from 0 to 100. Higher scores indicate better sexual quality of life. It has good psychometric properties [27–29]. The SQoL had been validated in Spanish and this version was utilized [30].

#### 2.2.4. Sexual Desire Inventory (SDI-2)

The SDI-2 is an 11-item self-report measure of sexual desire that assesses dyadic and solitary sexual desire as two subscales [31]. Participants rate how strong their desire would be in a variety of sexual situations during the last month. Scores are summed across items, with higher scores reflecting stronger sexual desire [32]. The scale has excellent psychometric properties and it has been used in the general population, in clinical samples, and had been validated in Spanish [33].

### 2.3. Procedure

Local researchers reviewed medical records to identify individuals with TBI who were treated at the Hospital Universitario Hernando Moncaleano Perdomo de Neiva, between October 2012 and June 2013 in Neiva. Each candidate was screened by telephone to determine whether he or she met inclusion or exclusion criteria. The healthy control group was recruited from the general population through flyers at neighborhood churches, stores, and restaurants and by general word of mouth. After the details of the study were explained to each eligible candidate, individuals who expressed interest were invited to participate. Once the individuals with TBI and healthy controls agreed to participate in the study, they were asked to sign a form that indicated their informed consent in accordance with regulations approved by Universidad Surcolombiana, Colombia. All of the participants completed an interview with a graduate student under the supervision of a university professor. The student collected demographic information and conducted a psychosexual evaluation with TBI survivors and the healthy controls. The interviews lasted for approximately 1 hour. This study was reviewed and approved by the ethics committee of Universidad Surcolombiana.

## 3. Results

**Figure 1:**
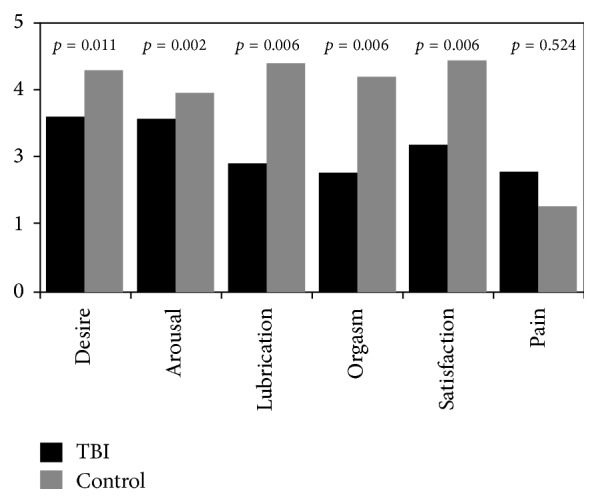
Female Sexual Function Index mean subscale scores.

Women with TBI reported significantly lower mean scores than the control group on the FSFI subscales of desire, arousal, lubrication, orgasm, and sexual satisfaction (see Figure 1). The mean overall FSFI score for women with TBI was 15.12, while it was 22.04 for the control group. 83% of the participants with TBI met the clinical cutoff for sexual dysfunction while 69% of the participants in the control group met the same criteria, although these differences were not statistically significant (*p* > 0.05).

Participants in the TBI group scored significantly lower on the SQoL and the SDI Dyadic subscale and notably higher than the women in the control group on the ISS. There were no statistically significant between-group differences with Overall SDI and the SDI Individual subscale scores. See Table 2.

Although the sample size for these regressions was limited to the 29 women with TBI in the current sample, multiple regressions with three predictors using this sample approximately meet the conventional guidelines of having 10 participants per predictor variable. Additionally, a power analysis was conducted for a multiple regression with three predictors. With an *α* = 0.05 and power (1 − *β*) = 0.80, the current study's sample size of 29 could uncover only large-sized effects (with an *f* ^2^ > 0.44). As a result, null omnibus results should be interpreted with caution. For the TBI group only, four simultaneous multiple regressions assessed the relationships between the variables of age, time since the injury (months), and injury severity as measured by GCS score at admission with the FSFI subscales of arousal, lubrication, orgasm, and satisfaction. The model for arousal was significant (*F*(3,25) = 8.028, *p* = 0.001, *R* ^2^ = 0.491) and accounted for 49% of the variability of the arousal subscale. Age (*β* = −0.443, *p* = 0.006) and months since injury (*β* = 0.472, *p* = 0.006) statistically significantly added the model, while GCS did not (*β* = −0.080, *p* = 0.613).

The model for the FSFI lubrication subscale was statistically significant (*F*(3,25) = 6.416, *p* = 0.002, *R* ^2^ = 0.435) and accounted for 44% of the variability of this subscale. The variables of age (*β* = −0.473, *p* = 0.005) and months since injury (*β* = 0.413, *p* = 0.018) were independently related to the lubrication subscale, but GCS score was not (*β* = 0.083, *p* = 0.619).

The third model with the orgasm subscale as the independent variable was statistically significant (*F*(3,25) = 7.887, *p* = 0.001, *R* ^2^ = 0.486) accounting for 49% of the variance in this variable. Within this model, the variables of age (*β* = −0.373, *p* = 0.018) and months since the injury (*β* = 0.563, *p* = 0.001) were uniquely related to orgasm functioning, whereas GCS was not (*β* = 0.759, *p* = 0.445).

The final model for sexual satisfaction was significant (*F*(3,25) = 8.835, *p* < 0.001, *R* ^2^ = 0.515) and accounted for 52% of the variance in this variable. Age (*β* = −0.415, *p* = 0.008) and months since injury (*β* = 0.544, *p* = 0.008) were independently associated with satisfaction, but GCS was not (*β* = 0.226, *p* = 0.150).

Also for the TBI group only, a series of simultaneous multiple regressions were performed in order to investigate the connections from age, months since injury, and TBI severity with sexual desire. The model with Overall SDI score as the dependent variable was statistically significant (*F*(3,25) = 7.913, *p* = 0.001, *R* ^2^ = 0.487) and accounted for 49% of the variability. Age negatively predicted overall sexual desire (*β* = −0.59, *p* < 0.001), though months since injury (*β* = 0.28, *p* = 0.081) and GCS (*β* = −0.747, *p* = 0.462) did not. In another regression, the same variables significantly accounted for 46% of the variability of the SDI Dyadic subscale (*F*(3,25) = 7.024, *p* = 0.001, *R* ^2^ = 0.457). Age was the only variable with a unique effect (*β* = −0.638, *p* < 0.001), as months since injury (*β* = 0.154, *p* = 0.313) and GCS (*β* = −0.040, *p* = 0.805) were not statistically significant.

Five multiple regressions were also conducted to assess whether the independent variables of age, months since injury, and initial GCS were associated with the dependent variables of SQOL, FSFI Pain, FSFI Desire, ISS, and SDI Individual scores. None of these models were statistically significant.

## 4. Discussion

The principal objectives of this research were to evaluate the sexual functioning, desire, and satisfaction in women with TBI, as well as compare these areas with a control group. Women with TBI reported sexual difficulties in the six areas of sexual functioning evaluated and fared worse than the control group in eight of eleven sexuality constructs measured.

### 4.1. Sexual Functioning

The mean FSFI score for women with TBI was 15.12, well below the 26.55 cutoff for sexual dysfunction [34]. In reference to the FSFI, there were statistically significant differences between women with TBI and the control group on five of the six subscales. Women with TBI reported less desire, arousal, sexual satisfaction, lubrication, and orgasm function. Similar results of decreased sexual functioning in control studies were found in Australia using the Brain Injury Questionnaire of Sexuality [10] and in the United States [8]. When taking age into account, these differences were exclusive to the 46–55 age range in the Australian study [10]; this contrasts with the results of the present research where between-group differences were found with a nearly one decade younger average age (36.41 TBI group; 36.34 control group) of the participants. The American team found differences between the study group and control in arousal and vaginal lubrication, as was found in this study. However, they found a higher frequency of pain during sexual activity which was not reflected in these data.

The multiple regressions found that age has a negative relationship with both arousal and lubrication in women with TBI; similar results have been found by other authors in population-based studies [35, 36] and in persons with TBI [8, 12, 16]. Conversely, months since injury had a positive relationship with these measures, such that sexual functioning may be improving over time. Sabhesan and Natarajan [36] reported that, without intervention, in twelve months after the incident, only 38% of people who had sustained a TBI returned to preincident sexual functioning. Ponsford et al. [11] found that shorter recovery time was related to lower sexual functioning. The findings of the present study suggest that the passage of time does correlate with improved sexual functioning in women with TBI. However, this does not fully resolve sexual dysfunction, perhaps indicating that sexuality-specific intervention programs may be beneficial toward recovering sexual functioning after TBI.

### 4.2. Sexual Satisfaction

Two measures for sexual satisfaction were utilized, the Female Sexual Functioning Index Satisfaction subscale and the Index of Sexual Satisfaction. For both measures, women with TBI reported significantly lower sexual satisfaction than their counterparts in the control group. This disparity could be related to the difficulties reported with desire, arousal, lubrication, and orgasm. Consistency in orgasm frequency has been found to influence sexual satisfaction in Spanish women without TBI [37] and may likely have played a role in the current sample. The multiple regression analyses utilizing the FSFI Satisfaction subscale found that age has a negative relationship with sexual satisfaction similar to results from other studies in general populations [38]. This relationship does not explain between-group differences; however, the average age of the study and control group participants was not statistically different. While age appears to be a factor in sexual satisfaction, it does not account for the lower reported sexual satisfaction levels in TBI survivors compared to the control group.

### 4.3. Desire

Of the two measures used to assess sexual desire, differences between the study groups were found with the FSFI Desire subscale and the Sexual Desire Inventory Dyadic subscale; women with TBI endorsed less desire than participants without. Similar differences were not found in a comparable control group method study [39]. Although no significant between-group differences were found in the SDI Individual subscale, women with TBI reported on average more desire for self-pleasure than women in the control group (see Table 2). Perhaps because of the many between-group differences outlined above, holistically, women with TBI reported overall lower sexual quality of life, as indicated by FSFI and SQoL scores. Altered sexual functioning after TBI may negatively impact one's sexual relationships, sexual confidence, and emotional well-being. Together, these results add to the literature documenting disparities in sexuality among women with TBI compared to the general population. In clinical settings, rehabilitation programs should pay specific attention to sexuality issues in order to improve quality of life after TBI. Further research would benefit from assessing possible physiological causes of sexual difficulties such as posttraumatic hypopituitarism, which has been found to be present in approximately 35% of people with moderate to severe TBI [40]. Other studies should use qualitative methods to further understand women's experiences. Unfortunately, to date, no standardized and empirically supported intervention exists for sexuality after TBI, and as a result, many of the disparities documented in the current study are going unaddressed by the rehabilitation community. The evaluation of sexuality-specific intervention programs is a critical next step in TBI rehabilitation.

### 4.4. Limitations

A limitation of this study is the relatively small sample size; therefore these results should be interpreted with caution. In fact, various effects should be interpreted with caution, due to the fact that familywise error corrections were not used because of the study's limited sample size. The cross-sectional design of this study is a potential limitation, because the results speak to a specific moment in time. Longitudinal methods would be required to verify whether the sexual difficulties and disparities in sexuality measures between women with TBI and control groups found are maintained over time. Preinjury sexual functioning, desire, and satisfaction were not assessed in this study; therefore, no comparison of the results can occur regarding pre- and postinjury sexuality. The participants of this study were recruited in a specific region of Colombia, where they did not have access to rehabilitation programs, as compared to many other studies [15]. This is both a limitation and an advantage of this study; these data only allow one to better understand the ramifications of moderate to severe TBI on sexuality when general and specific rehabilitation strategies have not been carried out.

Sexuality was assessed through self-report measures, and social desirability may play a role in such a sensitive and often stigmatized construct. Similarly, individuals with TBI frequently do not accurately perceive changes in their functioning levels [41], and anosognosia could have influenced these results. In order to avoid this possible limitation or reporting bias, studies could contrast self-report data with their partner's evaluation of sexual functioning. Other factors, which could influence sexual functioning, desire, and satisfaction, are self-esteem and fatigue [39], employment status and annual income [42], presence of endocrine disorders [2], lesion location [6], use of antidepressants [5], decreased social participation [15], and depression [11, 15] or partners' possible sexual difficulties, which were not evaluated in this project and should be considered in follow-up studies.

The participants with TBI all had moderate to severe TBI according to the GCS. These results cannot be extrapolated to people with mild TBI. GCS scores did not correlate with or predict any other variables. As indicated by Balestreri et al. [43], this measure does not have a high predictive value for outcomes. The results from this study imply that this measure also does not have a high predictive value for these sexuality measures.

## 5. Conclusions

This cross-sectional self-report control group design study gives insight into sexual functioning, desire, and satisfaction in women with TBI. Compared to the control group, women with TBI had reduced sexual desire, arousal, orgasm function, sexual satisfaction, and lubrication. No between-group differences were found with pain during sexual activity or overall sexual desire scores. TBI severity as evaluated by the GCS did not predict any of the variables, while age was negatively associated with arousal, lubrication, orgasm, and sexual satisfaction and months since injury positively influenced the same areas of sexuality. These findings suggest the critical need for sexual rehabilitation interventions for women with TBI.

## Figures and Tables

**Table 1:** Participant demographics.

Variable	TBI group	Control group	*p* value
	(*n* = 29)	(*n* = 29)	
Age, years, mean (SD)	36.41 (11.87)	36.34 (11.97)	NS
Education, years, mean (SD)	9.41 (3.35)	9.45 (3.33)	NS
Cause of TBI, %			
Motorcycle accident	44.8	—	
Interpersonal violence	13.8	—	
Fall	13.8	—	
Sports accident	10.3	—	
Pedestrian accident	6.9	—	
Car accident	6.9	—	
Other	3.4	—	
Relationship status, %			NS
Married	31.0	31.0	
Cohabiting	34.5	31.0	
Single	24.1	31.0	
Separated	6.9	6.9	
Widowed	3.4	—	
Sexual orientation, %			NS
Heterosexual	96.6	96.6	
Homosexual	3.4	3.4	
Employment status, %			NS
Full-time employment	34.5	31.0	
Unemployed	34.5	37.9	
Student	6.9	10.3	
Part-time employment	6.9	6.9	
Stay at home parent	6.9	3.4	
Other	10.3	6.9	

**Table 2:** Sexuality measure mean scores in the TBI (*n* = 29) and control (*n* = 29) groups and between-group differences.

	TBI group		Control group		*p* value
	Mean (M)	Standard deviation (SD)	Mean (M)	Standard deviation (SD)	
SQoL	56.54	16.56	84.64	13.00	*p* < 0.001
SDI^*∗*^	38.21	23.58	49.59	21.09	*p* = 0.135
SDI Dyadic^*∗*^	26.38	18.67	37.38	15.76	*p* = 0.027
SDI Individual^*∗*^	4.62	7.23	2.72	5.31	*p* = 0.346
ISS	47.44	14.75	22.61	8.96	*p* < 0.001

Notes: ^*∗*^Kolmogorov-Smirnov and Shapiro-Wilk; *p* < 0.05.

SQoL: Sexual Quality of Life Questionnaire; SDI: Sexual Desire Index; ISS: Index of Sexual Satisfaction.
